# The flame retardant DE-71 (a mixture of polybrominated diphenyl ethers) inhibits human differentiated thyroid cell function *in vitro*

**DOI:** 10.1371/journal.pone.0179858

**Published:** 2017-06-23

**Authors:** Thit Mynster Kronborg, Juliana Frohnert Hansen, Åse Krogh Rasmussen, Katrin Vorkamp, Claus Henrik Nielsen, Marie Frederiksen, Jacob Hofman-Bang, Christoffer Holst Hahn, Louise Ramhøj, Ulla Feldt-Rasmussen

**Affiliations:** 1 Department of Medical Endocrinology, Rigshospitalet, University of Copenhagen, København Ø, Denmark; 2 Department of Environmental Science, Aarhus University, Roskilde, Denmark; 3 Institute of Inflammation Research, Rigshospitalet, University of Copenhagen, København N, Denmark; 4 The National Research Center for the Working Environment, København Ø, Denmark; 5 Department of Nephrology, Rigshospitalet, University of Copenhagen, København Ø, Denmark; 6 Department of Ear Nose Throat Head and Neck Surgery, Rigshospitalet, København Ø, Denmark; 7 National Food Institute, Technical University of Denmark, Kongens Lyngby, Denmark; Hokkaido Daigaku, JAPAN

## Abstract

**Background:**

Normal thyroid function is essential for general growth and metabolism, but can be affected by endocrine disrupting chemicals (EDCs). Polybrominated diphenyl ethers (PBDEs) have been used worldwide to reduce flammability in different materials and are suspected to be EDCs. The production of the commercial Penta- and OctaBDE mixtures is banned, but DecaBDEs and existing products may leak PBDEs into the environment. Our aim was to investigate the effect of the PentaBDE mixture DE-71 on human thyroid cells *in vitro*.

**Materials and methods:**

Primary human thyroid cells were obtained as paraadenomatous tissue and cultured in monolayers. The influence of DE-71 on cyclic adenosine monophosphate (cAMP) and thyroglobulin (Tg) production was examined in the culture medium by competitive radioimmunoassay and enzyme-linked immunosorbent assay, respectively. Real-time quantitative PCR analysis of thyroid-specific genes was performed on the exposed cell cultures. PBDE concentrations were determined in cellular and supernatant fractions of the cultures.

**Results:**

DE-71 inhibited Tg-release from TSH-stimulated thyrocytes. At 50 mg/L DE-71, mean Tg production was reduced by 71.9% (range: 8.5–98.7%), and cAMP by 95.1% (range: 91.5–98.8%) compared to controls). Expression of mRNA encoding Tg, TPO and TSHr were significantly inhibited (p<0.0001, p = 0.0079, and p = 0.0002, respectively). The majority of DE-71 added was found in the cell fraction. No cytotoxicity was found.

**Conclusions:**

DE-71 inhibited differentiated thyroid cell functions in a two phase response manner and a concentration-dependent inhibition of Tg and cAMP production, respectively, as well as expression of mRNA encoding Tg, TPO and TSHr. Our findings suggest an inhibiting effect of PBDEs on thyroid cells.

## Introduction

Polybrominated diphenyl ethers (PBDEs) have been used as flame retardants to reduce flammability of electronic equipment, upholstery, textiles, plastics and building materials [1]. Commercial production of the PentaBDE and OctaBDE mixtures was banned in the European Union in 2004 and phased out in the USA in 2005 [1–4], due to increasing concerns of adverse physiological effects and persistence of the compounds [5–7]. Since 2009, the Penta- and OctaBDE mixtures have been registered as global persistent organic pollutants under the Stockholm Convention [8]. However, because of a long life-time PBDEs will continue to leak from products and accumulate in the environment and humans for many years [1;9]. DE-71 was one of the commercial PentaBDE mixtures, mainly consisting of the tetra-, penta-, and hexabrominated congeners [10]. The congener pattern of commercial PentaBDE mixtures is reflected in indoor dust and air, and the congeners are found in human samples, such as blood, placenta, amniotic fluid and breast milk [11;12].

The PBDEs are believed to be toxic at low concentrations [1]. Epidemiological studies indicate neurobehavioral and developmental effects as well as effects on the reproductive and endocrine system. Animal and *in vitro* studies have also shown effects of PBDEs on the immune system [13–15]. PBDEs are classified as possible endocrine disrupting chemicals (EDCs) [16;17]. Some EDCs are suspected to affect thyroid function, thereby resulting in thyroid homeostasis disturbances [18]. The thyroid gland produces the thyroid hormones thyroxine (T4) and triiodothyronine (T3), which are essential for growth, metabolism and brain development. The protein molecule thyroglobulin (Tg) in thyrocytes is precursor for thyroid hormone production. Thyroid peroxidase catalyses oxidation of iodine, which is necessary for correct thyroid hormone synthesis on the Tg precursor. However, many targets are available for possible disruption of thyroid function [18;19]. Hyper- as well as hypo-secretion of thyroid hormones affect multiple organ systems. Some PBDEs penetrate the placenta [20;21] and thereby may have a damaging effect on the development of neurological functions in embryos [22–24]. Moreover, children are particularly sensitive to changes in thyroid homeostasis [17] and more exposed than adults, since PBDEs are present in breast milk [1], and children likely have a larger uptake of PBDEs with dust [1;25] and an immature metabolic capacity [26]. Several epidemiologic studies in humans and wildlife suggest a correlation between PBDE exposure and altered thyroid homeostasis [13;26–29]. Neurodevelopmental effects resulting from PBDE exposure of children might have consequences for society, in terms of lower IQ levels and associated costs [27].

The direct effects of PBDEs on the thyroid gland are unknown, and we therefore aimed to study effects and cytotoxicity of the commercial mixture DE-71 on primary human thyroid cell function. This was investigated via analysis of protein secretion and gene expression in the differentiated thyroid cells. We hypothesised that PBDEs would disturb these functions.

## Materials and methods

### Cell cultures

Preparation of cells took place as previously described [30] with minor modifications. In summary, primary human thyroid cells were obtained as paraadenomatous tissue (assumed to be normal) from thyroidectomies performed at the department of Ear Nose Throat (ENT)-Head and Neck surgery, Rigshospitalet, University of Copenhagen. The thyroid tissue was cut into small pieces, and the cells were separated from connective tissue by incubation with collagenase I (Sigma-Aldrich, St. Louis, MO, USA) and dispase II (Roche, Basel, Switzerland) for 60–90 min. at 37°C. The digested tissue was filtered, culture medium was added and the suspension was centrifuged at 1200 x G for five min. The cells were cultured in 24-well plates containing HAM’s F-12 media (Panum Institute, Copenhagen University, Denmark) supplemented with 5% fetal bovine serum (FBS) (Biological Industries, Beit HaEmek, Israel), 1% L-glutamine (Panum Institute, Copenhagen University, Denmark), 1% non-essential amino acids (Gibco, Invitrogen, Carlsbad, CA, USA), 1% penicillin and streptomycin (Invitrogen), and six nutritional factors (6H media): 1 IU/L bovine thyroid stimulating hormone (TSH) (Sigma-Aldrich), human insulin; 10 mg/L (rhinsulin; Humulin, Eli Lilly), 6 mg/L transferrin (Sigma-Aldrich), 0.01 mg/L Gly-His-Lys acetate (Sigma-Aldrich), 10 mg/L somatostatin (Calbiochem), 10^−8^ M hydrocortisone (Calbiochem). Cells were grown until a confluent monolayer was visualized in the wells. The growth period lasted for a maximum of 10 days in order to avoid loss of properties of the cells [31]. The suitability of each cell culture to study cellular functions was ensured by assessing outcome variables from both TSH- and un-stimulated cells.

### DE-71 exposure

Cell monolayers were cultured for three additional days without TSH (5H medium) before stimulation and addition of DE-71 in serum free medium for 72 hours. DE-71 (pentaBDE, lot 7550K20A was kindly provided by Martha Axelstad, National Food Institute, Technical University of Denmark and Dr. Kevin Crofton of the U.S. Environmental Protection Agency), was dissolved in dimethyl sulfoxide (DMSO, D2438 Sigma-Aldrich, St. Louis, MO, USA) prior to dissolving in culture medium without FBS, resulting in final concentrations of 0.01, 0.1, 1, 5, 10 and 50 mg DE-71/L, respectively, and 1 ‰ DMSO in the cell cultures. Three negative controls (without DE-71) were included on each culture plate: one non-stimulated (5H medium) without DMSO and two TSH-stimulated (6H medium) cell populations, respectively, with and without 1 ‰ DMSO added. All experiments were conducted in duplicate in the culture plates. The cell function is dependent on TSH, which was assessed by comparing 6H and 5H controls. Cells were visually inspected by light microscopy at the end of incubation. Cell supernatants were harvested and stored at -20°C until analyses. The cells were harvested on ice, immediately after removal of the cell supernatant using lysis buffer (Qiagen, Hilden, Germany) and stored at -80°C with prior addition of 70% ethanol (350 μL pr. well).

### Cell function analysis

The function of the cells was evaluated by measuring the second messenger cyclic adenosine monophosphate (cAMP) and Tg concentrations in the cell supernatants. cAMP and Tg were analyzed as previously described by a competitive protein binding method detected by a radioimmunoassay (RIA) and an enzyme-linked immunosorbent assay (ELISA), respectively [30;32]. To assess cAMP production, 3-isobutyl-1-methylxanthine was diluted in ethanol (final ethanol concentration 1%) and added to the cultures concurrently with DE-71 solutions. The Tg assay was designed to measure Tg in culture medium and not for use in serum. Tg and cAMP analysis were each performed on seven cultures, representing seven patients, each on one 24-well plate. In total, tissue was obtained from 11 patients (nine females, two males, age: 20–82 years). Three tissue samples contained an amount of cells eligible for growth on two 24-well plates, why each of these three samples gave rise to one culture for Tg and one culture for cAMP analysis.

The intra-assay variation of the cAMP method was 4.9% at 0.5 μM and 3.2% at 1.57 μM and the inter-assay variation was 24.8% for the low control (range 0.38–0.66 μM) and 11.5% for the high control (range 1.34–1.77 μM) (n = four duplicates for each control). The calibration range was 0.05 to 2.0 μM.

The intra-assay variation of the Tg-analysis was 7.3% and 10.9% at 55 and 116 μg/L, respectively, and the inter-assay variation was 22.6% for the low control (range 32.3–63.5 μg/L) and 26.2% for the high control (74.7–149.1 μg/L) (n = six samples in duplicate for each type of control, see *DE-71 exposure*). The calibration range was 10 to 500 μg/L.

### Real-time quantitative polymerase chain reaction (RT-qPCR)

Cell function analysis was also carried out by determining the gene expression of Tg, thyroid peroxidase (TPO), sodium iodine symporter (NIS), thyroid stimulating hormone receptor (TSHr) and interleukin (IL)-6. IL-6 is secreted from human thyroid cells as shown earlier [33], but it is not expressed in differentiated thyrocytes and thus served as negative control.

Total RNA from harvested primary human thyroid cells from five cultures (cell remnants from cultures used for Tg analysis) was extracted with Rneasy Mini Kit (Qiagen) according to the manufacturer’s protocol. The concentration and purity of the achieved RNA was measured on a NanoDrop spectrophotometer (nd-1000, Wilmington, DE, USA). cDNA was synthesized (Superscript VILO synthesis kit (Invitrogen) by mixing four μL of the VILO reaction mix, two μL of the Superscript enzyme mix, the RNA from each sample and RNAase free water to a total volume of 20 μL. The volume of added RNA was adjusted to unify the final concentration of RNA in all samples from the same cell culture. Samples were incubated for 10 min. at 25°C, 60 min. at 50°C and 5 min. at 85°C, after which 80 μL of 0.5X Tris-EDTA-buffer (Sigma-Aldrich) were added. For real-time quantitative polymerase chain reaction (RT-qPCR), SYBR Green JumpStart Taq Ready Mix (Sigma-Aldrich) was used. Primers and their respective sequences are listed in Table 1. The primers were validated by SYBR melting curves and the qPCR fragment sizes were checked on agarose gels. A pool of undiluted cDNA was used for standards. To each reaction, 4 μL of the SYBR Green JumpStart Taq ReadyMix, 10 μL H_2_O and 1 μL primermix (1μM final concentration of each primer) were added. RT-qPCR was done on Lightcycler 480 II (Roche) with the following cycling: Initial denaturation at 94°C for two min., followed by 45 cycles of 30 seconds at 94°C, 45 seconds at 59°C and 1.5 min. at 72°C, and finally melting curve analysis. The high number of cycles was used due to very low expression of both NIS and IL-6. The specificity of the amplified products was verified by melting curve analysis and the relative mRNA quantification was achieved by standard curve method. The obtained gene of interest-expression was then divided by the housekeeping gene -expression of the same sample. Neither DE-71, TSH nor DMSO had any influence on these housekeeping genes. Using GENORM-calculation on the gene expression of B2M, GAPDH and ACTB, the last two were the most stable genes and the genes of interest were therefore normalized to the geometric mean of GAPDH and ACTB.

**Table 1:** Sequences of the primers used in RT-qPCR.

Gene	Direction	Sequence
Tg	forward	GGGCGGGCAGTCAGCAGAGAGTG
	reverse	CCATAGTGGGCAGCCTCGGGTGAG
TPO	forward	GGAGAGTGCTGGGATGGAAG
	reverse	GGATTTGCCTGTGTTTGGAA
SLC5A5 (NIS)	forward	ACCTTCTACACGGCTGTGGGCGGC
	reverse	CTCGGGTCAGGGTTAAAGTCCATG
TSHr	forward	GAATGCTTTTCAGGGACTATGCAAT
	reverse	ACAGCAGTGGCTTGGGTAAGAA
IL-6	forward	AGAGTAACATGTGTGAAAGCAGCAA
	reverse	CCTCAAACTCCAAAAGACCAGTGA
B2M	forward	TGTGCTCGCGCTACTCTCTC
	reverse	CTGAATGCTCCACTTTTTCAATTCT
ACTB	forward	CTGGAACGGTGAAGGTGACA
	reverse	AAGGGACTTCCTGTAACAACGCA
GAPDH	forward	CATGAGAAGTATGACAACAGCCT
	reverse	AGTCCTTCCACGATACCAAAGT

### Cytotoxicity

DE-71 induced cytotoxicity was analysed by assessing the content of lactate dehydrogenase (LDH) in cell culture supernatants as described previously [34]. For this purpose, a homogenous membrane integrity assay, CytoTox-ONE (Promega, Fitchburg, WI, USA), was used according to the manufacturer’s protocol with the modification that LDH was not measured directly in cell cultures but in harvested supernatants. Briefly, one experiment was performed with cell culture supernatants from an experiment containing single determinations of six DE-71 concentrations (0.01; 0.1; 1; 5; 10 and 50 mg DE-71/L) and duplicates of the negative controls (6H medium with and without 1‰ DMSO and 5H medium) and positive controls (0.02, 0.2 and 1.9 mg/L Triton X-100). The frozen cell supernatants were thawed and mixed by vortex. Fifty μL of each culture media sample were transferred to a 96-wells micro plate (Th.Geyer, Renningen, Germany) followed by addition of the same volume CytoTox-One reagent to all wells. The micro plate was mixed gently on a shaker and incubated at room temperature for 10–15 min. Twenty-five μl stop solution were added, and the plate was mixed again on a shaker before results were read on a fluorometer (Victor2, PerkinElmer, Waltham, MA, USA). The LDH content was expressed proportional to the produced fluorescence and given in relative fluorescence units. The cytotoxicity kit passed quality control assays according to the manufacturer.

### PBDE analysis

Three supernatants and three cell samples of pooled triplicates (addition of 0.01, 10 and 50 mg/L, respectively), one 1‰ DMSO control supernatant sample, one 1‰ DMSO control cell sample, three standard solutions of DE-71 in DMSO (0.01, 5 and 50 mg/L) and two solutions of DE-71 in cell media were analysed for content and composition of BDE-congeners at Aarhus University, Department of Environmental Science. This was done after the function analyses using once thawed samples. The measurements followed accredited methods for PBDEs in biota as described elsewhere [35] and included 11 tri- to heptabrominated congeners (BDE-17, 28, 47, 49, 66, 85, 99, 100, 153, 154 and 183). Inter-batch variation of the in-house reference material (n = 18), sand eel oil, ranged from 2.7% (BDE-47) to 14% (BDE-17), with a mean of 6.6% for all congeners. The instrumental detection limits ranged between 0.05 and 0.25 pg. Briefly, all samples (except for the three standard solutions of DE-71 in DMSO) were spiked with recovery standards, dried with diatomaceous earth (Varian) and Soxhlet extracted with hexane:acetone (4:1). The extracts were cleaned up on a multilayer column consisting of aluminum oxide, silica and acidified silica. After elution, volume reduction and addition of the internal standard (BDE-71, Cambridge Isotope Laboratories, Tewksbury, MA, USA), the samples were analysed by gas chromatography—mass spectrometry (GC-MS) with electron capture negative ionization. Quantification was based on two calibrations of ten standards each (0.05–25 ng/mL). The samples were extracted in three batches, each also containing 1–2 spiked control samples (approximately 10 ng of the individual congeners) and one blank. The three DE-71 stock solutions in DMSO, along with four spiked control samples (approximately 10 ng of the individual congeners) and two blanks, were evaporated to dryness in silicone vials [35], re-dissolved in iso-octane including the internal standard BDE-71 and analysed without further clean-up.

### Statistics

For analysis of cAMP and Tg, culture supernatants were assessed in duplicates from 11different patients (total cultures, n = 14). The Tg- and cAMP-productions (mean of duplicates) as well as gene expression (cells from five different patients, duplicates were pooled before RNA extraction) were analysed by two way ANOVA followed by Tukey’s honest significant difference post hoc analysis to compare experiment groups (R, version 3.1.2). Ln-transformed data was used when necessary. Results from the Tukey analysis are given as estimated differences between groups in tables.

The responses from TSH-stimulated cells with and without DMSO were compared by paired t-tests to investigate if DMSO had an isolated effect on stimulated thyroid cells. Appropriate responsiveness of the thyrocytes to TSH was demonstrated by comparing Tg and cAMP release from unstimulated (5H) and TSH-stimulated (6H) cells, respectively (paired t-test).

Values below limit of detection (LOD) in the Tg analysis were replaced by the lowest standard value in the assay while undetectably low single cAMP concentrations were replaced by half the cAMP level of lowest detectable value in the assay. These differences were performed according to the different assay methods. Results were considered statistically significant when p < 0.05.

### Ethics

The study was approved by The Danish committees on health research ethics, Capital region (Protocol number: H-1-2012-110 and additional protocol 44717). Paraadenomatous tissue samples were obtained from patients undergoing thyroidectomies. Patients gave oral and written informed consent prior to surgery. The paraadenomatous tissue was selected by the chief surgeon during the procedure.

## Results

### Quality control of the cells

Inspection of the cell cultures by light microscopy demonstrated that DE-71 formed intracellular black granula in a concentration-dependent manner, with most granula observed in the wells exposed to 50 mg DE-71/L and none in the controls. Cell confluence was not affected by DE-71 addition, indicating that viability was not affected. TSH-responsiveness with stimulation of the thyrocytes was ensured by consistent stimulation of cAMP when cultured in medium with TSH compared to medium without TSH (p = 0.029,).

### Tg production

No significant difference was found between the TSH-stimulated production of Tg by thyrocytes in DMSO- and culture media controls, respectively (p = 0.13). The DMSO-control (median = 88.3 μg Tg per L, range: 16.7–2,399 μg/L) was used as negative control in all further statistical calculations as DE-71 was dissolved in DMSO, thereby making DE-71 the only difference between control and experiment.

**Fig 1:**
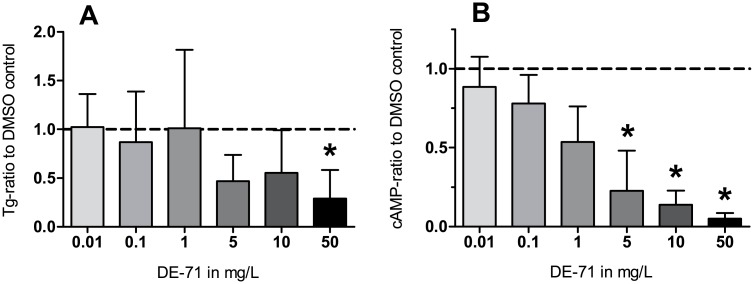
Influence of DE-71 on thyroglobulin (1A) and cAMP (1B) production by primary human thyrocytes. Primary human thyrocyte cultures were incubated with 1 IU/L TSH and **0.0**1–50 mg DE-71/L, dissolved in dimethyl sulfoxide (DMSO). After 72 hours, the concentration of thyroglobulin (Tg) and cAMP in the culture media was measured by ELISA and by radioimmunoassay, respectively, and expressed as a ratio to the Tg (A)/cAMP (B)-concentration found in cultures exposed to DMSO control devoid of DE-71. One cAMP experiment resulted in undetectable control values for both duplicates, and the experimental values in that culture were therefore not eligible for statistical analysis. The dotted line represents the ratio to the control = 1. Bars and error bars represent mean+SD of seven (A)/six (B) experiments performed in duplicates. *p<0.05 compared to DMSO control after post hoc analysis, see Table 2.

DE-71 displayed an inhibiting two-phase -response effect on the TSH-induced Tg production (Fig 1A) (p<0.0001). Post hoc analysis (Table 2) showed that cultures exposed to 50 mg DE-71/L contained significantly less Tg than cultures exposed to either 0, 0.01, 0.1 or 1 mg DE-71/L (p = 0.0002–0.0012).

**Table 2:** Tukey honest significance difference post hoc analysis of ln-transformed thyroglobulin (Tg)-data; Tg-production form TSH-stimulated cells, non-significant differences not shown, p-value comparing two different DE-71 concentrations or DE-71 against control, *Estimated Ratio*: *estimate of individual difference/interceptin Tg production between different DE-71 concentration exposures (groups) in TukeyHSD*, *converted by exponential function*, *CI*: *confidence interval*.

DE-71 Exposure Comparisons, Tg data	p-value	Estimated Ratio (95% CI)
50 mg DE-71/L vs. control	**0.0002**	0.19 (0.07; 0.5)
50 mg DE-71/L vs. 0.01 mg DE-71/L	**0.0003**	0.19 (0.07; 0.5)
50 mg DE-71/L vs. 0.1 mg DE-71/L	**0.0024**	0.24 (0.09; 0.7)
50 mg DE-71/L vs. 1 mg DE-71/L	**0.0012**	0.22 (0.08; 0.6)

### cAMP production

As shown in Fig 1B, DE-71 also caused inhibition of the TSH-induced production of cAMP, in a concentration-dependent manner (p<0.0001). The post hoc analysis (Table 3) showed that cultures exposed to 5, 10 and 50 mg DE-71/L contained less cAMP than the DMSO controls, and significant inhibitions were also found when comparing cAMP production in presence of DE-71 at a concentration of 50 mg DE-71/L with that observed in presence of 0.01–10 mg DE-71/L (p<0.0001–0.025).

**Table 3:** Tukey honest significance difference post hoc analysis of ln-transformed cyclic adenosine monophosphate (cAMP) data; cAMP-production form TSH-stimulated cells, non-significant differences not shown, p-value comparing two different DE-71 concentrations or DE-71 against control, *cAMP*: *cyclic adenosine monophosphate*, *Estimated Ratio*: *estimate of individual difference/intercept in cAMP production between different DE-71 concentration exposures (groups) in TukeyHSD*, *converted by exponential function*, *CI*: *confidence interval*.

DE-71 Exposure Comparisons, cAMP data	p-value	Estimated Ratio (95% CI)
5 mg DE-71/L vs. control	**0.019**	0.13 (0.022; 0.73)
10 mg DE-71/L vs. control	**0.007**	0.11 (0.020; 0.64)
50 mg DE-71/L vs. control	**<0.0001**	0.019 (0.003; 0.11)
5 mg DE-71/L vs. 0.01 mg DE-71/L	**0.025**	0.15 (0.026; 0.85)
10 mg DE-71/L vs. 0.01 mg DE-71/L	**0.014**	0.13 (0.023; 0.74)
50 mg DE-71/L vs. 0.010 mg DE-71/L	**<0.0001**	0.022 (0.004; 0.13)
5 mg DE-71/L vs. 0.1 mg DE-71/L	**0.043**	0.17 (0.030; 0.96)
10 mg DE-71/L vs. 0.1 mg DE-71/L	**0.025**	0.15 (0.026; 0.85)
50 mg DE-71/L vs. 0.1 mg DE-71/L	**<0.0001**	0.025 (0.004; 0.14)
50 mg DE-71/L vs. 1 mg DE-71/L	**<0.0001**	0.038 (0.007; 0.22)
50 mg DE-71/L vs. 5 mg DE-71/L	**0.025**	0.15 (0.026; 0.85)
50 mg DE-71/L vs. 10 mg DE-71/L	**0.043**	0.17 (0.030; 0.96)

### RT-qPCR

Comparisons of the negative controls without DE-71 with and without TSH showed no significant differences for the expression of any gene (Table 4). Median values of all examined thyroid-specific genes were higher in TSH-stimulated thyrocytes than in unstimulated controls. The production of IL-6, on the other hand, was apparently not affected by TSH as expected.

**Table 4:** Medians and ranges of gene expression for cell culture media controls, n = number of cultures, p-value comparing 5H gene levels to 6H gene levels, medians (ranges) for unstimulated controls without TSH and stimulated controls with TSH, respectively. *Tg*: *thyroglobulin*, *TSHr*: *thyroid stimulating hormone receptor*, *TPO*: *thyroid peroxidase*, *IL-6*: *interleukin 6*, *NIS*: *sodium iodide symporter*.

Gene	n	p-value	Median (range) of quantified mRNA normalized to housekeeping gene from unstimulated control, 5H (Control without TSH)	Median (range) of quantified mRNA noramlized to housekeeping gene from stimulated control, 6H (Control with TSH)
Tg	5	0.058	0.49 (0.32; 0.95)	1.15 (0.70; 2.00)
TSHr	5	0.14	0.73 (0.20; 1.43)	1.10 (0.91; 2.43)
TPO	5	0.071	0.31 (0.14; 0.88)	1.21 (0.80; 2.35)
IL-6	5	0.33	10.03 (5.18; 22.90)	8.71 (3.68; 17.99)
NIS	4	0.28	2.95 (1.14; 4.81)	5.34 (3.75; 17.32)

**Fig 2:**
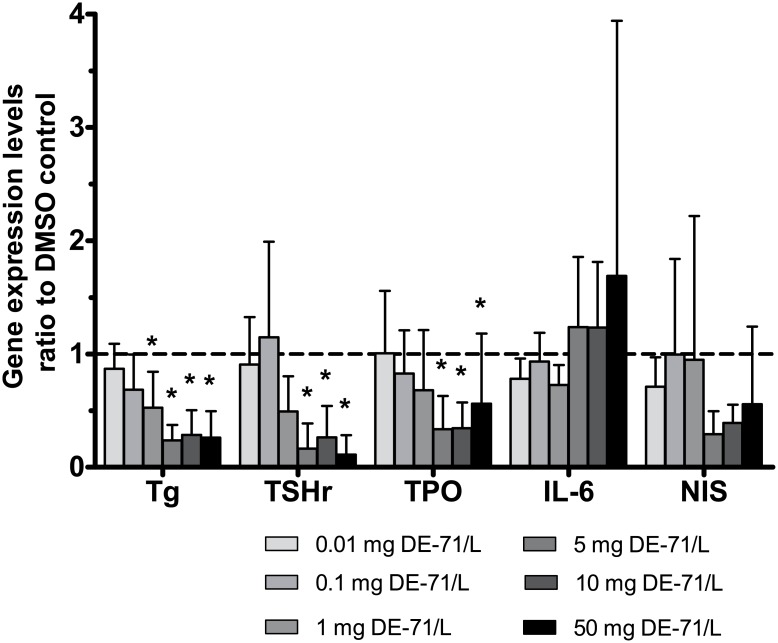
Influence of the flame retardant DE-71 on thyroid specific gene expression. Cultures of human thyrocytes were stimulated with 1 IU/TSH and in absence or presence of DE-71, at different concentrations. After 72 hours, expression of thyroglobulin (Tg), thyroid stimulated hormone receptor (TSHr), thyroid peroxidase (TPO), interleukin-6 (IL-6) and sodium iodide symporter (NIS) genes were measured by RT-qPCR. Shown are mean+SD of the ratio of quantified mRNA normalized to housekeeping gene to the negative control (control = 1; dotted line) for five experiments. *p<0.05 for the null-hypothesis that DE-71 had no effect.

Expression of Tg-mRNA, TPO-mRNA and TSHr-mRNA was inhibited by DE-71 (p<0.0001, p = 0.0029 and p = 0.00014, respectively), while DE-71 did not influence IL-6-mRNA nor NIS-mRNA production with statistical significance according to two-way ANOVA and the post hoc analysis (Fig 2, Table 5). The pattern shown in Fig 2 demonstrates a two phase response for Tg, TSHr and TPO gene expression. Even though no statistical significant difference was found for inhibition of NIS, the pattern in Fig 2 is similar for NIS compared to Tg, TSHr and TPO.

**Table 5:** ANOVA and Tukey honest significant difference post hoc results from RT-qPCR analysis, n = number of cultures, p-value comparing gene expression between individual different DE-71 concentrations, statistically significant differences between the indicated groups are shown. All other comparisons were not statistically significantly different and values are not shown. *HSD*: *honest significant difference*, *Estimated difference*: *estimate of individual difference/intercept in gene expression between the indicated DE-71 concentration exposures (groups) in TukeyHSD*.

Gene	n	p-value ANOVA	Transformed data used	Significant p-value Tukey	Groups compared	Estimated difference (95% CI) Tukey
Tg	5	**<0.0001**	no	0.0091	Control– 1 mg/L DE-71	-0.98(-1.78;-0.19)
				0.00012	Control– 5 mg/L DE-71	-1.42 (-2.22;-0.63)
				0.00022	Control– 10 mg/L DE-71	-1.36 (-2.15;-0.56)
				0.00024	Control– 50 mg/L DE-71	-1.35 (-2.14;-0.55)
				0.0025	0.01–5 mg/L DE-71	-1.11 (-1.91;-0.32)
				0.0046	0.01–10 mg/L DE-71	-1.05 (-1.84;-0.25)
				0.0051	0.01–50 mg/L DE-71	-1.04 (-1.84;-0.25)
TPO	5	**0.0029**	no	0.013	Control– 5 mg/L DE-71	-1.18 (-2.17;-0.19)
				0.013	Control– 10 mg/L DE-71	-1.18 (-2.17;-0.18)
				0.046	0.01–5 mg/L DE-71	-1.01 (-2.00;-0.01)
				0.047	0.01–10 mg/L DE-71	-1.00 (-2.00;-0.01)
TSHr	5	**0.00014**	no	0.0081	Control– 5 mg/L DE-71	-1.46 (-2.62;-0.29)
				0.018	Control– 10 mg/L DE-71	-1.33 (-2.50;-0.16)
				0.0044	Control– 50 mg/L DE-71	-1.55 (-2.72;-0.38)
				0.028	0.01–50 mg/L DE-71	-1.26 (-2.43;-0.09)
				0.0053	0.1–5 mg/L DE-71	-1.52 (-2.69;-0.35)
				0.012	0.1–10 mg/L DE-71	-1.39 (-2.56;-0.23)
				0.0029	0.1–50 mg/L DE-71	-1.61 (-2.78;-0.45)
IL-6	5	0.73	ln	-	-	-
NIS	4	0.13	ln	-	-	-

The post hoc-analysis revealed an influence from various DE-71 concentrations on Tg-, TPO- and TSHr gene expression (Table 5). Thus, cultures exposed to 1–50 mg DE-71/L expressed less Tg than DMSO controls. Significant differences were also found between 0.01 mg DE-71/L and 5–50 mg DE-71/L. The changes in Tg protein and mRNA levels correlated well with each other.

Cultures exposed to 5–10 mg and 5–50 mg DE-71/L displayed significantly lower expression of TPO and TSHr, respectively, than controls. Moreover, less TPO-expression occurred in presence of 10 mg DE-71/L than in presence of 0.01 mg DE-71/L. TSHr expression was lower in presence of 50 mg/L than in presence of 0.01 mg/L DE-71, and also lower in presence of 5–50 mg DE-71/L than in presence of 0.1 mg DE-71/L (p-values are listed in Table 5).

### Cytotoxicity

**Fig 3:**
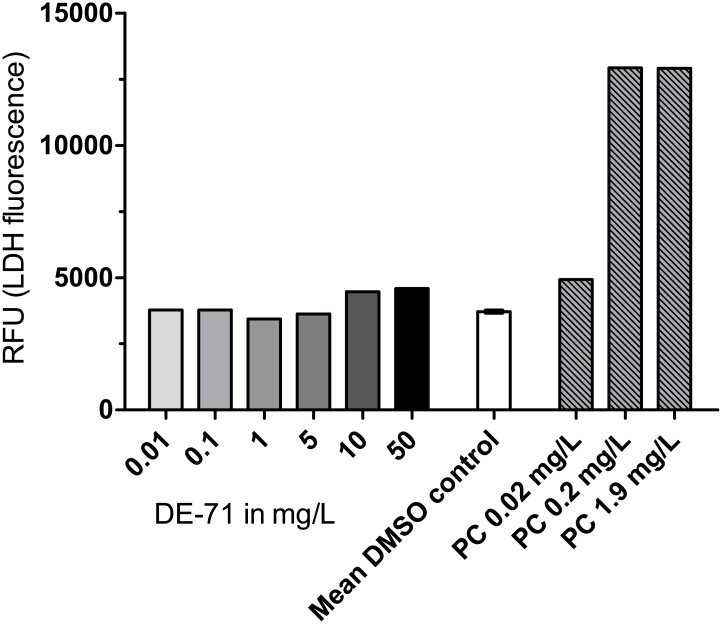
Cytotoxicity of DE-71 in primary human thyrocytes; Lactate dehydrogenase (LDH) release from human thyrocytes after DE-71 addition at six concentrations. LDH was measured in relative fluorescence units (RFU), n = 1 culture; *conc*.: *concentration*, *DMSO*: *dimethyl sulfoxide*, *PC = positive control (Triton X-100)*.

We next examined the cytotoxic effect of DE-71. To this end, cultures exposed to Triton X-100 served as positive control. Additions of 0.02, 0.2 or 1.9 mg/L of Triton X-100 caused increases in LDH concentration of the culture medium compared to the negative DMSO-control (Fig 3). However, only cells exposed to 0.2 and 1.9 mg/L Triton X-100 resulted in visibly affected cells by light microscopy. The cells had loosened from the vials and were fewer in number. The analyses were done on cell supernatants, which might include cell fragments, but adherent cell fractions were not included in the analysis.

At concentrations of 50 mg DE-71/L and 10 mg DE-71/L, DE-71 appeared to have some cytotoxic effect, indicated by an increase in supernatant LDH concentration compared to the DMSO control. However, the induced LDH release was lower than that induced by the lowest positive Triton X-100 control.

### PBDE analysis

The concentration of PBDE congeners in the supernatants and in the cells was close to added concentrations (Table 6). The vast majority of DE-71 added to thyrocyte cultures was found in the cell remnants. Thus, the concentration of DE-71 in cell supernatants, measured as the concentration of BDE-99, was only 0.2–1.8% of the added DE-71 concentration, while concentrations of DE-71 in cell remnants were 95.1–110% of the added concentrations (Table 6). The analyses of controls containing DMSO showed traces of BDE-99 (0.13 μg/L).

**Table 6:** Concentrations of DE-71 in supernatants and cells. Measured concentration of PBDE congeners (μg/L) in cells and supernatants in comparison to added concentrations of DE-71. *LOD*: *Limit of detection*.

DE-71 congeners	0.01 mg DE-71/L		1 mg DE-71/L		50 mg DE-71/L
	Supernatant	Cells	Supernatant	Cells	Supernatant
**BDE-28**	< LOD	< LOD	< LOD	0.94	< LOD
**BDE-47**	0.059	1.90	0.161	230	33.4
**BDE-49**	< LOD	< LOD	< LOD	4.02	0.715
**BDE-66**	< LOD	0.0930	< LOD	4.81	1.00
**BDE-85**	< LOD	0.240	< LOD	19.6	6.29
**BDE-99**	0.646	6.31	0.507	496	146
**BDE-100**	< LOD	1.21	0.0758	93.7	24.8
**BDE-153**	< LOD	0.972	0.148	56.3	26.4
**BDE-154**	< LOD	0.692	0.0636	44.8	17.7
**ΣPBDE**	0.124	11.4	0.955	951	257
**ΣPBDE**	11.5		951		49,532

The DE-71 stock solutions mainly contained BDE-99 (50%), BDE-47 (29%), BDE-100 (9%), BDE-153 (5.1%) and BDE-154 (3.7%) (see [36]This pattern was similar in the supernatant and cells, however, with relatively more BDE-47 in the cells (23 ± 5.9%) than in the supernatant (13 ± 3.0%), and relatively less BDE-154 and BDE-153.

The average recovery of the extracts and DE-71 standards was 90% (range: 75–105%) and 89% (range: 79–103%), respectively. One of the blanks contained traces of PBDEs at the level of the detection limits. The PBDEs spiked to co-extracted control samples were recovered at 97% on average (n = 8), ranging from 91% for BDE-183 to 100% for BDE-66.

## Discussion

An influence of EDCs on thyroid gland function has been suggested [18]. In the present study, we examined the influence of the PentaBDE mixture DE-71 on cultures of human primary thyroid cells grown in the presence or absence of TSH. We have previously shown that such cultures contain 98% Tg-producing cells, confirming the thyroid cell identity [30], and that TSH consistently increases cAMP release [37]. In this study we used Tg protein concentrations as surrogate marker of the ability of the cultured thyroid cells to secrete T3 and T4. The actual ability of the cell to produce the hormones is rather low, as previously found by our group (unpublished results). This is probably due to absence of iodine in the culture media, to the use of the cells in a non-polarized way [38] or due to cell arrangement in a monolayer compared to colloid rich follicles. Thyroid cell arrangement is different in vitro compared to in vivo (monolayer compared to colloid rich follicles). We therefore did not include measurements of these hormones in the present experimental set-up.

The main congeners of DE-71 were determined by GC-MS-analysis and were in agreement with the profile of DE-71 specified by La Guardia et al. [10]. The GC-MS analysis confirmed that the amount of PBDEs added to the assays was available in the cell cultures, i.e. no major loss occurred through e.g. adsorption or degradation.

Of key importance, we found that DE-71 inhibited the thyrocyte production of Tg in a two phase response manner and cAMP in a concentration-dependent manner. Furthermore, the expression of mRNA encoding Tg, TPO, and TSHr was also reduced by DE-71. Tg protein production and Tg gene expression seemed to be inhibited in a parallel manner. By contrast, expression of NIS was not significantly affected by DE-71, but appeared inhibited in a pattern similar to the inhibition of Tg, TPO and TSHr supporting the general inhibiting tendency of DE-71. However, the expression levels of NIS were low at baseline, why there is an uncertainty related to the expression levels of NIS. As expected, the expression of IL-6 was insignificant confirming identity of the cells as differentiated thyrocytes. As previously shown, IL-6 levels are expected to be lower in the TSH-stimulated control than in the unstimulated control [39].

The absolute change in gene expression may appear modest and this should be taken into account. Some of this could be due to the large between culture variation in human primary thyroid cell cultures [30]. However, the gene expression results are supported by the decreased protein levels found in the Tg and cAMP analysis which brings biological significance to the affected gene expression pathways.

The mechanisms by which absorbed PBDEs act on the human thyroid homeostasis have not been clarified [19;24]. Suggestions include induction of uridine diphosphate glucuronyltransferase (UDPGT) activity [28;29], direct thyroid tissue attack, alterations in transport of thyroid hormones and hormone deactivation [16]. Furthermore, the chemical structure of PBDEs is very similar to the structure of the thyroid hormone T4 [13]. Thus, a competitive effect of the PBDEs on the thyroid hormone receptor is suspected, but the potential of such a mechanism is unknown [40]. Hence, multiple thyroid related endpoints impacted by PBDE exposure probably exist. No previous research has investigated the direct effects of DE-71 on thyrocytes. The combination of inhibited gene expression and protein production found in this study supports the hypothesis that DE-71 inhibits thyrocyte function by direct thyroid attack. This inhibition could be compared to that of the proinflammatory cytokine IL-1β. IL-1β was used as a positive control as it has consistently been shown to inhibit the differentiated function of thyroid cells (reviewed in [41]) in the same assay as the one in the present study [31;39;42;43]. Though direct comparison between previous studies and the present DE-71 study is not possible, since cells from different individuals have been used, the grade for DE-71 inhibition shown here is comparable to the one induced by IL-1β, albeit possibly at higher concentrations for DE-71 indicating a lower potency of DE-71 compared to IL-1β. However, the grade of DE-71 inhibition shown here seems to be as potent to the inhibition induced by IL-1β, at least for Tg and cAMP secretion, and Tg-, TPO- and TSHr-gene expression.

In summary, multiple studies in other model systems have found DE-71 to be thyrotoxic. Overall the mode of action is presumed to be a combination of induction of liver enzymes and displacement of thyroid hormones from serum transport proteins together affecting the excretion of thyroid hormones [16;28;29]. As these pathways are not present in this assay the effect of DE-71 on the thyrocytes could be through different mechanisms, potentially adding a new mode of action to DE-71.

Correlations between thyroid disturbances and PBDEs in humans have been described epidemiologically. One study showed significantly higher PBDE levels in umbilical cord blood and breast milk samples from mothers with a history of thyroid disease compared to healthy mothers [44], and another study suggested a correlation between congenital hypothyroidism and PBDEs in the serum [45], while Jacobson et al. found higher TSH and lower T4 concentrations among toddlers [46]. In 2016 Oulhote et al. found a positive association between reported hypothyroidism in women (aged 30–79 years) and the sum of PBDEs and BDE-47 alone in serum, however not significant for other BDEs [47]. Other disturbances in thyroid hormone concentrations correlating to PBDE-levels have been described as well [48;49]. This indicates an increased risk for disease in presence of higher blood PBDE-levels. Our findings support this notion. However, the causality of the correlation was questioned by Eggesbø et al. [50], who did not observe an association between TSH concentrations in whole blood of neonates and the PBDE concentrations in their mothers’ milk. In addition, Julander et al. found no significant increases in plasma PBDE concentrations with increasing length of exposure in a high-risk group (workers at an electronic recycling facility), and no significant correlations to thyroid hormone-concentrations [51]. They did, however, find a tendency to negative associations between free T4 and serum levels of BDE-28, BDE-153 and BDE-183. In conclusion, epidemiological studies have not given consistent results, as the studies have used varying endpoints, exposures and methods.

More consistent results have been obtained from animal studies. Several *in vivo* rodent studies have pointed towards a thyroid hormone disturbance after PBDE-exposure, where lowered concentrations of T4 were the most frequent and significant finding [28;29;52–55]. Three of six studies found no changes in T3 levels in mice and rats [28;29;54], three studies measured TSH [28;54;55] where only one found an effect, and only in male rats [55]. However, all animal studies found significantly reduced T4 levels after exposure to PBDE. In addition de-Miranda et al. found decreased T4-serum concentrations in postnatal rats exposed to DE-71 with a reversible effect by adding T4 [56].

In line with our findings, another *in vitro* study revealed inhibited thyroid hormone-dependent Purkinje cell development after addition of DE-71 in two doses (10^−10^ M and 10^−8^ M) in the developing rat cerebellum [57].

In general, PBDE-doses used in animal experiments and measured exposure levels are difficult to compare with the concentrations of this study due to differences in units, the number of included congeners and method differences [1;12;58–60]. Our experiments were performed in serum-free conditions and thus the free PBDE concentrations may be lower *in vivo* where serum binding proteins for PBDE are present. The concentration range of DE-71 used in this study were similar to former *in vitro* studies with PBDEs [61;62]. The lowest concentration of DE-71 used in our study was 0.01 mg DE-71/L. As BDE-47 accounted for 29% of DE-71, this resulted in a concentration of BDE-47 of 2.9 μg/L, which is approximately 10–20 times higher than a typical median concentration of BDE-47 in serum of the US population. The levels in the general population of Europe are about 10 times lower than the US body burdens [1;12]. However, the distributions are usually highly skewed with medians clearly lower than arithmetic means, reflecting higher internal exposure levels in some individuals, which likely are closer to the concentrations used in this study. Animal experiments showing responses of the thyroid systems tend to use doses above that of this study [e.g. [55]], but rarely determine serum concentrations. The exposure doses usually exceed estimates of human exposure by several orders of magnitude [63].

In view of the above, our *in vitro* results should be extrapolated to *in vivo* actions with caution. There are a limited number of target sites and toxicity pathways in cells *in vitro* compared to the amount of target sites in an organ/multi-organ system, with many interactions and feedback systems [64]. In addition, it is not known, whether DE-71 accumulates intracellularly, which is possible since half-life ranges are long and PBDEs are highly lipophilic. The congener assessment showed orders of magnitude lower concentrations in the cell supernatants after ended experiments. Afterwards, we recovered the DE-71 concentrations added to cells in the respective cell remnants of 95–110%, which is an almost complete recovery. This indicated an intracellular accumulation of DE-71 or adhesion of DE-71 to the cells. Our measurements did not distinguish between cellular uptake and attachment to cells. Due to the high lipophilicity of DE-71, some of the compound may have attached to glassware and instruments during storage and experiments, resulting in lower effective DE-71 concentrations than assumed. This was, however, contradicted by the experiments recovering almost all of the added DE-71. Hence, the observed effects on thyroid cell function were probably due to intracellular actions of DE-71.

Thyrocytes leaked LDH at high DE-71 concentrations indicating a cytotoxic effect of DE-71. However, all LDH-levels from DE-71 stimulated cell supernatants were below the lowest positive Triton X-100 control, and the cells appeared viable by microscopy. Hence, we assumed that the applied concentrations of DE-71 were not toxic to thyrocytes. However, it is possible that DE-71 has cytotoxic effects at low levels, which might explain the tendency of a no-dose dependent effect at higher DE-71 concentrations. It is highly relevant for future studies to examine this issue e.g. by other methods.

Although production of PBDEs is now prohibited and some studies have indicated stagnations in exposure levels and in human and animal sera [1], the results reported here remain of relevance, since the long half-lives of PBDEs and their continuous release from existing products may affect humans for many years [9]. Moreover, DecaBDE is still in production and can be degraded to lower brominated diphenyl ethers [65;66]. New chemicals replacing the old PBDEs are largely untested and may have the same chemical properties as the old ones including toxicity and persistence [67].

## Conclusion

DE-71 exerted a two-phase response of Tg and a concentration-dependent inhibition of cAMP production from human thyrocytes as well as inhibition of the expression of the genes encoding Tg, TSHr and TPO. Former human epidemiological studies found changed thyroid hormone levels following exposure to DE-71, while animal studies found reduced T4-concentrations. Our findings suggest an inhibiting effect of PBDEs on thyroid cells, which may explain the disturbances of thyroid homeostasis found by others. Further investigations regarding thyroid disturbances exerted by PBDEs in humans are needed. In addition, further experiments are needed at a lower concentration spectrum of DE-71 or individual PBDE congeners. To predict systemic toxicological effects, *in vivo* studies with an *in vivo* design are needed.

## References

[1] Frederiksen M, Vorkamp K, Thomsen M, Knudsen LE. Human internal and external exposure to PBDEs—a review of levels and sources. Int J Hyg Environ Health 2009 3;212(2):109–34. doi: 10.1016/j.ijheh.2008.04.005 18554980 10.1016/j.ijheh.2008.04.005

[2] BSEF, 2015. 2015. Bromine Science and Environmental Forum, http://www.bsef.com/ (July 16, 2015).

[3] Birnbaum LS, Staskal DF. Brominated flame retardants: cause for concern? Environ Health Perspect 2004 1;112(1):9–17. 14698924 10.1289/ehp.6559 PMC1241790

[4] de Wit CA, Herzke D, Vorkamp K. Brominated flame retardants in the Arctic environment—trends and new candidates. Sci Total Environ 2010 7 1;408(15):2885–918. doi: 10.1016/j.scitotenv.2009.08.037 19815253 10.1016/j.scitotenv.2009.08.037

[5] Darnerud PO, Eriksen GS, Johannesson T, Larsen PB, Viluksela M. Polybrominated diphenyl ethers: occurrence, dietary exposure, and toxicology. Environ Health Perspect 2001 3;109 Suppl 1:49–68. 11250805 10.1289/ehp.01109s149 PMC1240542

[6] de Wit CA. An overview of brominated flame retardants in the environment. Chemosphere 2002 2;46(5):583–624. 11999784 10.1016/s0045-6535(01)00225-9

[7] Zhang ZL, Leith C, Rhind SM, Kerr C, Osprey M, Kyle C, et al Long term temporal and spatial changes in the distribution of polychlorinated biphenyls and polybrominated diphenyl ethers in Scottish soils. Sci Total Environ 2014 1 15;468–469:158–64. doi: 10.1016/j.scitotenv.2013.08.029 24012902 10.1016/j.scitotenv.2013.08.029

[8] United Nations. Stockholm Convention on Persistant Organic Pollutants, Adoption of Amendments to annexes A, B and C, Reference C.N.524.2009.TREATIES-4 (Depositary Notification). 2009.

[9] Geyer HJ, Schramm K-W, Darnerud PO, Aune M, Feicht EA, Fried KW, et al Terminal elimination half-lives of the brominated flame retardants TBBPA, HBCD, and lower brominated PBDEs in humans. Organohalogen Compounds 2004 66, 3820–3825.

[10] LaA Guardia MJ, Hale RC, Harvey E. Detailed polybrominated diphenyl ether (PBDE) congener composition of the widely used penta-, octa-, and deca-PBDE technical flame-retardant mixtures. Environ Sci Technol 2006 10 15;40(20):6247–54. 17120549 10.1021/es060630m

[11] Stapleton HM, Eagle S, Anthopolos R, Wolkin A, Miranda ML. Associations between polybrominated diphenyl ether (PBDE) flame retardants, phenolic metabolites, and thyroid hormones during pregnancy. Environ Health Perspect 2011 10;119(10):1454–9. doi: 10.1289/ehp.1003235 21715241 10.1289/ehp.1003235 PMC3230439

[12] Vorkamp K, Thomsen M, Frederiksen M, Pedersen M, Knudsen LE. Polybrominated diphenyl ethers (PBDEs) in the indoor environment and associations with prenatal exposure. Environ Int 2011 1;37(1):1–10. doi: 10.1016/j.envint.2010.06.001 20609475 10.1016/j.envint.2010.06.001

[13] Kim YR, Harden FA, Toms LM, Norman RE. Health consequences of exposure to brominated flame retardants: a systematic review. Chemosphere 2014 7;106:1–19. doi: 10.1016/j.chemosphere.2013.12.064 24529398 10.1016/j.chemosphere.2013.12.064

[14] Lv QY, Wan B, Guo LH, Zhao L, Yang Y. In vitro immune toxicity of polybrominated diphenyl ethers on murine peritoneal macrophages: apoptosis and immune cell dysfunction. Chemosphere 2015 2;120:621–30. doi: 10.1016/j.chemosphere.2014.08.029 25462306 10.1016/j.chemosphere.2014.08.029

[15] Martin PA, Mayne GJ, Bursian FS, Tomy G, Palace V, Pekarik C, et al Immunotoxicity of the commercial polybrominated diphenyl ether mixture DE-71 in ranch mink (Mustela vison). Environ Toxicol Chem 2007 5;26(5):988–97. 17521147 10.1897/06-246r.1

[16] Darnerud PO. Brominated flame retardants as possible endocrine disrupters. Int J Androl 2008 4;31(2):152–60. doi: 10.1111/j.1365-2605.2008.00869.x 18315715 10.1111/j.1365-2605.2008.00869.x

[17] Legler J, Brouwer A. Are brominated flame retardants endocrine disruptors? Environ Int 2003 9;29(6):879–85. doi: 10.1016/S0160-4120(03)00104-1 12850103 10.1016/S0160-4120(03)00104-1

[18] Boas M, Feldt-Rasmussen U, Main KM. Thyroid effects of endocrine disrupting chemicals. Mol Cell Endocrinol 2012 5 22;355(2):240–8. doi: 10.1016/j.mce.2011.09.005 21939731 10.1016/j.mce.2011.09.005

[19] Boas M, Feldt-Rasmussen U, Skakkebaek NE, Main KM. Environmental chemicals and thyroid function. Eur J Endocrinol 2006 5;154(5):599–611. doi: 10.1530/eje.1.02128 16645005 10.1530/eje.1.02128

[20] Frederiksen M, Thomsen C, Froshaug M, Vorkamp K, Thomsen M, Becher G, et al Polybrominated diphenyl ethers in paired samples of maternal and umbilical cord blood plasma and associations with house dust in a Danish cohort. Int J Hyg Environ Health 2010 7;213(4):233–42. doi: 10.1016/j.ijheh.2010.04.008 20471317 10.1016/j.ijheh.2010.04.008

[21] Frederiksen M, Vorkamp K, Mathiesen L, Mose T, Knudsen LE. Placental transfer of the polybrominated diphenyl ethers BDE-47, BDE-99 and BDE-209 in a human placenta perfusion system: an experimental study. Environ Health 2010;9:32 doi: 10.1186/1476-069X-9-32 20598165 10.1186/1476-069X-9-32 PMC2908602

[22] Patel J, Landers K, Li H, Mortimer RH, Richard K. Thyroid hormones and fetal neurological development. J Endocrinol 2011 4;209(1):1–8. doi: 10.1530/JOE-10-0444 21212091 10.1530/JOE-10-0444

[23] Peltier MR, Klimova NG, Arita Y, Gurzenda EM, Murthy A, Chawala K, et al Polybrominated diphenyl ethers enhance the production of proinflammatory cytokines by the placenta. Placenta 2012 9;33(9):745–9. doi: 10.1016/j.placenta.2012.06.005 22749501 10.1016/j.placenta.2012.06.005 PMC3423505

[24] Zoeller RT. Environmental chemicals impacting the thyroid: targets and consequences. Thyroid 2007 9;17(9):811–7. doi: 10.1089/thy.2007.0107 17956155 10.1089/thy.2007.0107

[25] Fromme H, Becher G, Hilger B, Volkel W. Brominated flame retardants—Exposure and risk assessment for the general population. Int J Hyg Environ Health 2016 1;219(1):1–23. doi: 10.1016/j.ijheh.2015.08.004 26412400 10.1016/j.ijheh.2015.08.004

[26] Kim UJ, Oh JE. Tetrabromobisphenol A and hexabromocyclododecane flame retardants in infant-mother paired serum samples, and their relationships with thyroid hormones and environmental factors. Environ Pollut 2014 1;184:193–200. doi: 10.1016/j.envpol.2013.08.034 24060738 10.1016/j.envpol.2013.08.034

[27] Bellanger M, Demeneix B, Grandjean P, Zoeller RT, Trasande L. Neurobehavioral deficits, diseases, and associated costs of exposure to endocrine-disrupting chemicals in the European Union. J Clin Endocrinol Metab 2015 4;100(4):1256–66. doi: 10.1210/jc.2014-4323 25742515 10.1210/jc.2014-4323 PMC4399309

[28] Zhou T, Ross DG, DeVito MJ, Crofton KM. Effects of short-term in vivo exposure to polybrominated diphenyl ethers on thyroid hormones and hepatic enzyme activities in weanling rats. Toxicol Sci 2001 5;61(1):76–82. 11294977 10.1093/toxsci/61.1.76

[29] Zhou T, Taylor MM, DeVito MJ, Crofton KM. Developmental exposure to brominated diphenyl ethers results in thyroid hormone disruption. Toxicol Sci 2002 3;66(1):105–16. 11861977 10.1093/toxsci/66.1.105

[30] Rasmussen AK, Kayser L, Perrild H, Brandt M, Bech K, Feldt-Rasmussen U. Human thyroid epithelial cells cultured in monolayers. I. Decreased thyroglobulin and cAMP response to TSH in 12-week-old secondary and tertiary cultures. Mol Cell Endocrinol 1996 2 5;116(2):165–72. 8647316 10.1016/0303-7207(95)03711-x

[31] Krogh RA, Bech K, Feldt-Rasmussen U, Poulsen S, Holten I, Ryberg M, et al Interleukin-1 affects the function of cultured human thyroid cells. Allergy 1988 8;43(6):435–41. 2847577 10.1111/j.1398-9995.1988.tb00915.x

[32] Madsen SN, Badawi I, Skovsted L. A simple competitive protein-binding assay for adenosine-3',5'-monophosphate in plasma and urine. Acta Endocrinol (Copenh) 1976 1;81(1):208–14. 174364 10.1530/acta.0.0810208

[33] Diamant M, Kayser L, Rasmussen AK, Bech K, Feldt-Rassmussen U. Interleukin-6 production by thyroid epithelial cells. Enhancement by interleukin-1. Autoimmunity 1991;11(1):21–6. 1812993 10.3109/08916939108994704

[34] Hansen JF, Nielsen CH, Brorson MM, Frederiksen H, Hartoft-Nielsen ML, Rasmussen AK, et al Influence of Phthalates on in vitro Innate and Adaptive Immune Responses. PLoS One 2015;10(6):e0131168 doi: 10.1371/journal.pone.0131168 26110840 10.1371/journal.pone.0131168 PMC4482536

[35] Vorkamp K, Nielsen F, Kyhl HB, Husby S, Nielsen LB, Barington T, et al Polybrominated diphenyl ethers and perfluoroalkyl substances in serum of pregnant women: levels, correlations, and potential health implications. Arch Environ Contam Toxicol 2014 7;67(1):9–20. doi: 10.1007/s00244-013-9988-z 24435476 10.1007/s00244-013-9988-z

[36] Mynster KT, Frohnert HJ, Nielsen CH, Ramhoj L, Frederiksen M, Vorkamp K, et al Effects of the commercial flame retardant mixture DE-71 on cytokine production by human immune cells. PLoS One 2016;11(4):e0154621 doi: 10.1371/journal.pone.0154621 27128973 10.1371/journal.pone.0154621 PMC4851365

[37] Krogh Rasmussen Å, Kayser L, Perrild H, Brandt M, Bech K, Feldt-Rasmussen U. Human thyroid epithelial cells cultures in monolayers. II. Influence of serum on thyroglobulin and cAMP production. Molecular and Cellular Endocrinology 1996 116, 173–179. 8647317 10.1016/0303-7207(95)03712-8

[38] Ericson LE, Nilsson M. Structural and functional aspects of the thyroid follicular epithelium. Toxicol Lett 1992 12;64–65 Spec No:365–73. 10.1016/0378-4274(92)90209-3 1471192

[39] Rasmussen AK, Diamant M, Blichert-Toft M, Bendtzen K, Feldt-Rasmussen U. The effects of interleukin-1beta (IL-1beta) on human thyrocyte functions are counteracted by the IL-1 receptor antagonist. Endocrinology 1997 5;138(5):2043–8. doi: 10.1210/endo.138.5.5099 9112403 10.1210/endo.138.5.5099

[40] Sanders JM, Chen LJ, Lebetkin EH, Burka LT. Metabolism and disposition of 2,2',4,4'- tetrabromodiphenyl ether following administration of single or multiple doses to rats and mice. Xenobiotica 2006 1;36(1):103–17. doi: 10.1080/00498250500485107 16507516 10.1080/00498250500485107

[41] Rasmussen AK, Bendtzen K, Feldt-Rasmussen U. Thyrocyte-interleukin-1 interactions. Exp Clin Endocrinol Diabetes 2000;108(2):67–71. doi: 10.1055/s-2000-5797 10826510 10.1055/s-2000-5797

[42] Hansen JF, Brorson MM, Boas M, Frederiksen H, Nielsen CH, Lindstrom ES, et al Phthalates Are Metabolised by Primary Thyroid Cell Cultures but Have Limited Influence on Selected Thyroid Cell Functions In Vitro. PLoS One 2016;11(3):e0151192 doi: 10.1371/journal.pone.0151192 26985823 10.1371/journal.pone.0151192 PMC4795645

[43] Rasmussen AK. Cytokine actions on the thyroid gland. Dan Med Bull 2000 4;47(2):94–114. 10822801

[44] Kim UJ, Lee IS, Kim HS, Oh JE. Monitoring of PBDEs concentration in umbilical cord blood and breast milk from Korean population and estimating the effects of various parameters on accumulation in humans. Chemosphere 2011 10;85(3):487–93. doi: 10.1016/j.chemosphere.2011.08.008 21890170 10.1016/j.chemosphere.2011.08.008

[45] Kim UJ, Kim MY, Hong YH, Lee DH, Oh JE. Assessment of impact of internal exposure to PBDEs on human thyroid function—comparison between congenital hypothyroidism and normal paired blood. Environ Sci Technol 2012 6 5;46(11):6261–8. doi: 10.1021/es2038678 22578177 10.1021/es2038678

[46] Jacobson MH, Barr DB, Marcus M, Muir AB, Lyles RH, Howards PP, et al Serum polybrominated diphenyl ether concentrations and thyroid function in young children. Environ Res 2016 8;149:222–30. doi: 10.1016/j.envres.2016.05.022 27228485 10.1016/j.envres.2016.05.022 PMC4907865

[47] Oulhote Y, Chevrier J, Bouchard MF. Exposure to polybrominated diphenyl ethers (PBDEs) and hypothyroidism in canadian women. J Clin Endocrinol Metab 2016 2;101(2):590–8. doi: 10.1210/jc.2015-2659 26606679 10.1210/jc.2015-2659

[48] Chevrier J, Harley KG, Bradman A, Gharbi M, Sjodin A, Eskenazi B. Polybrominated diphenyl ether (PBDE) flame retardants and thyroid hormone during pregnancy. Environ Health Perspect 2010 10;118(10):1444–9. doi: 10.1289/ehp.1001905 20562054 10.1289/ehp.1001905 PMC2957927

[49] Han G, Ding G, Lou X, Wang X, Han J, Shen H, et al Correlations of PCBs, DIOXIN, and PBDE with TSH in children's blood in areas of computer E-waste recycling. Biomed Environ Sci 2011 4;24(2):112–6. doi: 10.3967/0895-3988.2011.02.004 21565681 10.3967/0895-3988.2011.02.004

[50] Eggesbo M, Thomsen C, Jorgensen JV, Becher G, Odland JO, Longnecker MP. Associations between brominated flame retardants in human milk and thyroid-stimulating hormone (TSH) in neonates. Environ Res 2011 8;111(6):737–43. doi: 10.1016/j.envres.2011.05.004 21601188 10.1016/j.envres.2011.05.004 PMC3143212

[51] Julander A, Karlsson M, Hagstrom K, Ohlson CG, Engwall M, Bryngelsson IL, et al Polybrominated diphenyl ethers—plasma levels and thyroid status of workers at an electronic recycling facility. Int Arch Occup Environ Health 2005 8;78(7):584–92. doi: 10.1007/s00420-005-0627-5 15902483 10.1007/s00420-005-0627-5

[52] Bondy GS, Lefebvre DE, Aziz S, Cherry W, Coady L, Maclellan E, et al Toxicologic and immunologic effects of perinatal exposure to the brominated diphenyl ether (BDE) mixture DE-71 in the Sprague-Dawley rat. Environ Toxicol 2013 4;28(4):215–28. doi: 10.1002/tox.20713 21544923 10.1002/tox.20713

[53] Fowles JR, Fairbrother A, Baecher-Steppan L, Kerkvliet NI. Immunologic and endocrine effects of the flame-retardant pentabromodiphenyl ether (DE-71) in C57BL/6J mice. Toxicology 1994 1 26;86(1–2):49–61. 8134923 10.1016/0300-483x(94)90052-3

[54] Kodavanti PR, Coburn CG, Moser VC, MacPhail RC, Fenton SE, Stoker TE, et al Developmental exposure to a commercial PBDE mixture, DE-71: neurobehavioral, hormonal, and reproductive effects. Toxicol Sci 2010 7;116(1):297–312. doi: 10.1093/toxsci/kfq105 20375078 10.1093/toxsci/kfq105

[55] Stoker TE, Laws SC, Crofton KM, Hedge JM, Ferrell JM, Cooper RL. Assessment of DE-71, a commercial polybrominated diphenyl ether (PBDE) mixture, in the EDSP male and female pubertal protocols. Toxicol Sci 2004 3;78(1):144–55. doi: 10.1093/toxsci/kfh029 14999130 10.1093/toxsci/kfh029

[56] de-Miranda AS, Kuriyama SN, da-Silva CS, do-Nascimento MS, Parente TE, Paumgartten FJ. Thyroid hormone disruption and cognitive impairment in rats exposed to PBDE during postnatal development. Reprod Toxicol 2016 8;63:114–24. doi: 10.1016/j.reprotox.2016.05.017 27233481 10.1016/j.reprotox.2016.05.017

[57] Kingsley I, Noriyuki K. DE71 suppresses thyroid hormone-mediated dendritogenesis and neuritogenesis in the developing cerebellum. Niger J Physiol Sci 2012;27(2):123–8. 23652225

[58] Streets SS, Henderson SA, Stoner AD, Carlson DL, Simcik MF, Swackhamer DL. Partitioning and bioaccumulation of PBDEs and PCBs in Lake Michigan. Environ Sci Technol 2006 12 1;40(23):7263–9. 17180976 10.1021/es061337p

[59] Wei RG, Zhao YX, Liu PY, Qin ZF, Yan SS, Li Y, et al Determination of environmentally relevant exposure concentrations of polybrominated diphenyl ethers for in vitro toxicological studies. Toxicol In Vitro 2010 6;24(4):1078–85. doi: 10.1016/j.tiv.2010.03.015 20362048 10.1016/j.tiv.2010.03.015

[60] Wu JP, Luo XJ, Zhang Y, Luo Y, Chen SJ, Mai BX, et al Bioaccumulation of polybrominated diphenyl ethers (PBDEs) and polychlorinated biphenyls (PCBs) in wild aquatic species from an electronic waste (e-waste) recycling site in South China. Environ Int 2008 11;34(8):1109–13. doi: 10.1016/j.envint.2008.04.001 18504055 10.1016/j.envint.2008.04.001

[61] Koike E, Yanagisawa R, Takigami H, Takano H. Brominated flame retardants stimulate mouse immune cells in vitro. J Appl Toxicol 2013 12;33(12):1451–9. doi: 10.1002/jat.2809 22972382 10.1002/jat.2809

[62] Wirth JR, Peden-Adams MM, White ND, Bossart GD, Fair PA. In vitro exposure of DE-71, a penta-PBDE mixture, on immune endpoints in bottlenose dolphins (*Tursiops truncatus*) and B6C3F1 mice. J Appl Toxicol 2014 4 7. 10.1002/jat.3008 24706408

[63] Harrad S, Hazrati S, Ibarra C. Concentrations of polychlorinated biphenyls in indoor air and polybrominated diphenyl ethers in indoor air and dust in Birmingham, United Kingdom: implications for human exposure. Environ Sci Technol 2006 8 1;40(15):4633–8. 16913117 10.1021/es0609147

[64] Groothuis FA, Heringa MB, Nicol B, Hermens JL, Blaauboer BJ, Kramer NI. Dose metric considerations in in vitro assays to improve quantitative in vitro-in vivo dose extrapolations. Toxicology 2015 6 5;332:30–40. doi: 10.1016/j.tox.2013.08.012 23978460 10.1016/j.tox.2013.08.012

[65] Christiansson A, Eriksson J, Teclechiel D, Bergman A. Identification and quantification of products formed via photolysis of decabromodiphenyl ether. Environ Sci Pollut Res Int 2009 5;16(3):312–21. doi: 10.1007/s11356-009-0150-4 19360447 10.1007/s11356-009-0150-4

[66] Lagalante AF, Shedden CS, Greenbacker PW. Levels of polybrominated diphenyl ethers (PBDEs) in dust from personal automobiles in conjunction with studies on the photochemical degradation of decabromodiphenyl ether (BDE-209). Environ Int 2011 7;37(5):899–906. doi: 10.1016/j.envint.2011.03.007 21458859 10.1016/j.envint.2011.03.007

[67] Dodson RE, Perovich LJ, Covaci A, Van den Eede N, Ionas AC, Dirtu AC, et al After the PBDE phase-out: a broad suite of flame retardants in repeat house dust samples from California. Environ Sci Technol 2012 12 18;46(24):13056–66. doi: 10.1021/es303879n 23185960 10.1021/es303879n PMC3525011

